# mCSM-membrane: predicting the effects of mutations on transmembrane proteins

**DOI:** 10.1093/nar/gkaa416

**Published:** 2020-05-29

**Authors:** Douglas E V Pires, Carlos H M Rodrigues, David B Ascher

**Affiliations:** Computational Biology and Clinical Informatics, Baker Institute, Melbourne, Victoria 3004, Australia; Structural Biology and Bioinformatics, Department of Biochemistry and Molecular Biology, Bio21 Institute, University of Melbourne, Parkville, VIC, 3052, Australia; School of Computing and Information Systems, University of Melbourne, Parkville, VIC, 3052, Australia; Computational Biology and Clinical Informatics, Baker Institute, Melbourne, Victoria 3004, Australia; Structural Biology and Bioinformatics, Department of Biochemistry and Molecular Biology, Bio21 Institute, University of Melbourne, Parkville, VIC, 3052, Australia; Computational Biology and Clinical Informatics, Baker Institute, Melbourne, Victoria 3004, Australia; Structural Biology and Bioinformatics, Department of Biochemistry and Molecular Biology, Bio21 Institute, University of Melbourne, Parkville, VIC, 3052, Australia; Department of Biochemistry, University of Cambridge, Cambridge, CB2 1GA, UK

## Abstract

Significant efforts have been invested into understanding and predicting the molecular consequences of mutations in protein coding regions, however nearly all approaches have been developed using globular, soluble proteins. These methods have been shown to poorly translate to studying the effects of mutations in membrane proteins. To fill this gap, here we report, mCSM-membrane, a user-friendly web server that can be used to analyse the impacts of mutations on membrane protein stability and the likelihood of them being disease associated. mCSM-membrane derives from our well-established mutation modelling approach that uses graph-based signatures to model protein geometry and physicochemical properties for supervised learning. Our stability predictor achieved correlations of up to 0.72 and 0.67 (on cross validation and blind tests, respectively), while our pathogenicity predictor achieved a Matthew's Correlation Coefficient (MCC) of up to 0.77 and 0.73, outperforming previously described methods in both predicting changes in stability and in identifying pathogenic variants. mCSM-membrane will be an invaluable and dedicated resource for investigating the effects of single-point mutations on membrane proteins through a freely available, user friendly web server at http://biosig.unimelb.edu.au/mcsm_membrane.

## INTRODUCTION

Integral membrane proteins play an essential role as the gateway to the cell, mediating transport, signalling and adhesion amongst many other functions. Mutations in membrane proteins are associated with a wide variety of common diseases, including heart disease, and consequently have been the site of action for over 50% of small molecule drugs (1). While they represent 20–30% of the genes in the human genome (2–4), they can be challenging to experimentally characterise as they tend to be unstable when extracted from the lipid bilayer. Consequently, less than 0.5% of experimentally determined structures are of integral membrane proteins.

There is therefore an increasing demand for methods capable of identifying mutations that might improve stability, to facilitate structural and functional characterization, and to identify novel disease-causing variants. Increasing computational power offers new opportunities to address these challenges, however most tools have been built using experimental information on predominantly globular, soluble proteins, and that have been shown to poorly translate to predicting the effects of mutations in membrane proteins (5).

The need for methods tailored for investigating mutation effects on transmembrane proteins becomes evident when considering the differences in residue environment in comparison with globular proteins. While many studies involving globular proteins have shown that solvent accessibility and residue depth correlates with mutation effects (6), for example buried and deep residues tend to be more conserved and mutations tend to have larger effects in stability, these might not be applicable for integral membrane proteins. To circumvent this, sophisticated ways to describe and represent residue environments are necessary.

We have previously tackled this task by developing the concept of graph-based signatures and showed they can provide powerful insights into understanding and predicting the effects of mutations on protein structures, including how mutations alter protein stability (6–8), dynamics (8), interactions with other molecules (7–14) and their relation to emergence of genetic diseases (15–27) and drug resistance (10,19,28–38).

Here we introduce mCSM-membrane, a web server that adapts and optimizes our well-established mCSM graph-based signatures framework in order to provide improved predictive performance of the molecular consequences of mutations in membrane proteins.

## MATERIALS AND METHODS

### Data sets

**Figure 1.**
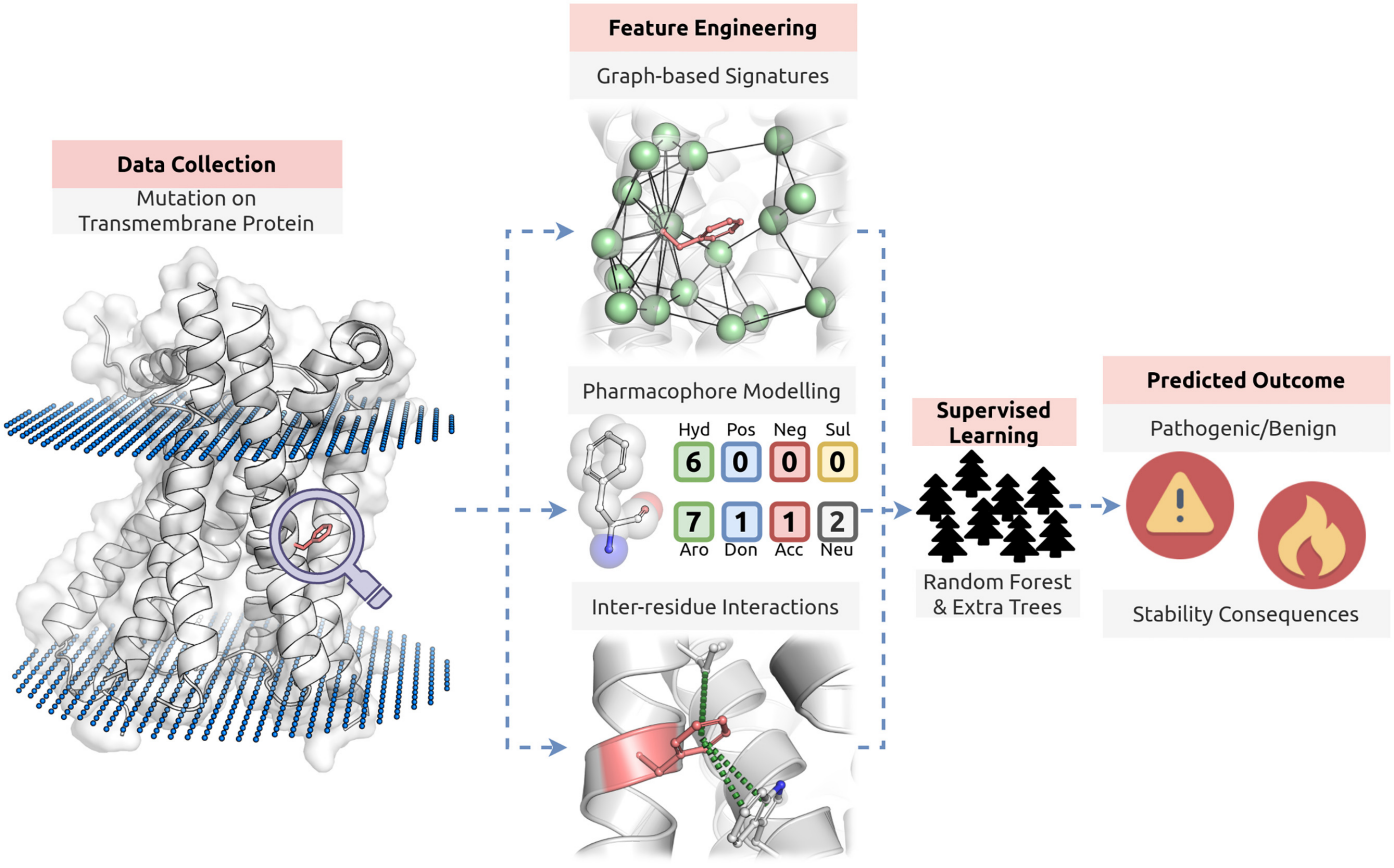
mCSM-membrane workflow. The first methodological step on mCSM-membrane was data collection. Experimentally validated effects of mutations on protein stability and pathogenicity were obtained for transmembrane proteins with available structures. During feature engineering, three main classes of features are generated: (i) graph-based signatures of the wild-type residue environment, (ii) a pharmacophore modelling of mutation effects (together with sequence-based properties) and (iii) the inter-residue interactions established. These are then used as evidence to train and test supervised learning algorithms. Random Forest for classification and Extra Trees for regression were the best performing and, therefore, selected methods.

The general workflow of mCSM-membrane is shown in Figure 1. mCSM-membrane was trained using two separate data sets of experimentally characterized mutations in transmembrane proteins, for which 3D structures were available.

The first data set contained experimentally measured effects of mutations on protein stability. This was obtained from (5) and encompasses 223 single-point missense mutations on 7 different proteins with experimental crystal structures available in the Protein Data Bank. The mutation effects were obtained in terms of the difference in Gibbs free energy of folding (ΔΔ*G* = Δ*G*_WT_ – Δ*G*_MT_, in Kcal/mol), with negative values denoting destabilising mutations and positive values denoting stabilising mutations, consistent with previously published methods. As discussed in previous works (8,10,13,14), the original data set was biased towards destabilising mutations (Supplementary Figure S1), which tend to affect machine learning methods. To circumvent this sampling limitation, we have modelled the hypothetic reverse mutations via comparative homology modelling and assigned the same ΔΔ*G* value as the forward mutation, with the opposite signal, in other words: ΔΔ*G*_WT→MT_ = –ΔΔ*G*_MT→WT_. Only reverse mutations with a measured effect in stability <2 kcal/mol were considered, in order to avoid situations where the reverse mutation could potentially compromise protein folding. Structures for reverse mutations were generated using the mutate function within Modeller (39) followed by refinement. A total of 181 reverse mutations were modelled, leading to a final data set of 404 mutations with associated stability effects (Supplementary Figure S1). Forward and reverse mutations pairs were kept together either in training or test sets. This was further divided into training (342 missense mutations occurring in 4 proteins, PDB IDs 2XOV, 1PY6, 3GP6 and 1QD6; 156 decreasing stability (ΔΔ*G* < −0.4 kcal/mol), 56 neutral, 130 increasing stability (ΔΔ*G* > 0.4 kcal/mol) and independent blind test (62 mutations occurring in the remaining three proteins, PDB IDs 1QJP, 2K73 and 1AFO, 28 decreasing stability, 14 neutral, 20 increasing stability). Training and test sets used in mCSM-membrane were non-redundant in terms of protein identity (<16% sequence identity – Supplementary Table S1) The proteins were also assessed in terms of their structural similarity using TMAlign and shared no more than 64% similarity.

The second data set was selected in order to train a structure-based model for predicting disease-associated mutations tailored for transmembrane proteins and was collected from (40). It comprises 539 single-point missense mutations in 62 different proteins, labelled either as benign or pathogenic, from the UniProtKB/Swiss-Prot variant database (41) This dataset was also further divided in training set (485 mutations, 347 pathogenic, 138 benign) and independent blind test (54 mutations, 38 pathogenic, 16 benign) for validation purposes, consistent with the data set defined by the BORODA-TM method for comparison purposes. Seven mutations described in the original data set, on two different residues of protein 4ZWJ could not be mapped to the structure available and therefore were removed from the training set. These compose non-redundant datasets, with sequence identity levels less than 50% and less than 75% structural similarity (calculated using TMalign).

The data sets used to develop mCSM-membrane are available to download at http://biosig.unimelb.edu.au/mcsm_membrane/data.

### Modelling effects of mutations

Single-point mutations can lead to a range of structural and functional changes. To try to encapsulate and explore the effects of single-point mutations on membrane proteins, we used two classes of structural features, in addition to sequence-based calculations.

### Graph-based structural signatures

One of the core components of mCSM-membrane is our well-established approach of using the concept of graph-based structural signatures (mCSM) to represent the environment of the wild-type residue (7) and describe both its geometry and physicochemical properties. Our approach aims to model wild-type residue environments as graphs, where atoms are represented as nodes (labelled based on their properties, i.e. pharmacophores) and their interactions as edges. By varying a distance cut off, different graphs are induced and cumulative distributions of distances for different pharmacophore/interactions generated, composing a concise and effective representation of the residue environment. This information is then used as evidence to train and test predictive methods using supervised learning.

### Molecular interactions

To capture information on whether, and how, a single-point mutation disrupted the intricate molecular interaction network, intra-molecular interactions were calculated using Arpeggio (42).

### Pharmacophore modelling and sequence-based features

The effect of the mutation on the residue environment is modeled using a pharmacophore representation for residues as previously described (7). Sequence-based features describing protein properties and amino acid composition were also calculated using the BioPython python library (43). These include AAindex amino acid mutation matrices and indexes representing physicochemical properties (44) and ProtParam, for calculating general protein sequence properties, including amino acid composition, molecular weight, isoelectric point, and hydropathicity (45).

Differently from globular proteins, neither residue depth, nor solvent accessibility, showed a significant correlation with stability effects (*r* = 0.07 and *r* = 0.09, respectively. Supplementary Figure S2).

## WEB SERVER

We have implemented mCSM-membrane as a user-friendly and freely available web server (http://biosig.unimelb.edu.au/mcsm_membrane/). The Bootstrap framework version 3.3.7 was used to develop the server front end, while the back-end was built in Python using the Flask framework version 1.0.2. The server is hosted on a Linux server running Apache 2.

### Input

mCSM-membrane can be used in two different ways: to either assess the effects of mutations on membrane protein stability, or to assess their pathogenicity (Supplementary Figure S3). For user-specified variations two options are available. The ‘Single Mutation’ option requires users to provide a PDB file or PDB accession code of the structure of the protein, the point mutation specified as a string containing the wild-type residue one-letter code, its corresponding residue number (consistent with the provided structure) and the mutant residue one-letter code. Alternatively, the ‘Mutation List’ option allows users to upload a list of mutations in a file for batch processing. For both options, users are also required to specify the chain identifier in which the wild-type residues are located as well as the Uniprot accession code for the protein of interest or provide its sequence in FASTA format. For homo-oligomers, mCSM-membrane will only consider the mutation in the provided chain, however the overall environment (oligomer) will be considered for feature generation.

In order to assist users to submit their jobs for predictions, sample submission entries are available in both submission pages and a help page is also available via the top navigation bar.

### Output

For the Stability option, mCSM-membrane outputs the predicted change in membrane protein stability (in kcal/mol), while for the Pathogenicity option mCSM-membrane outputs whether the mutation is predicted as Benign or Pathogenic.

With the Single Mutation option, mCSM-membrane outputs the prediction along with an interactive 3D viewer showing the wildtype residue environment and a depiction of the predicted transmembrane topology using Protter (46) (Supplementary Figure S4). In addition, all non-covalent interactions, generated using Arpeggio, made by the wildtype residue are available for download as a Pymol session file. For the Mutation List option, the results are summarized in a downloadable table from which users can access details for each single variant (Supplementary Figure S5).

## VALIDATION

### Predicting effects of mutations on transmembrane protein stability

**Figure 2.**
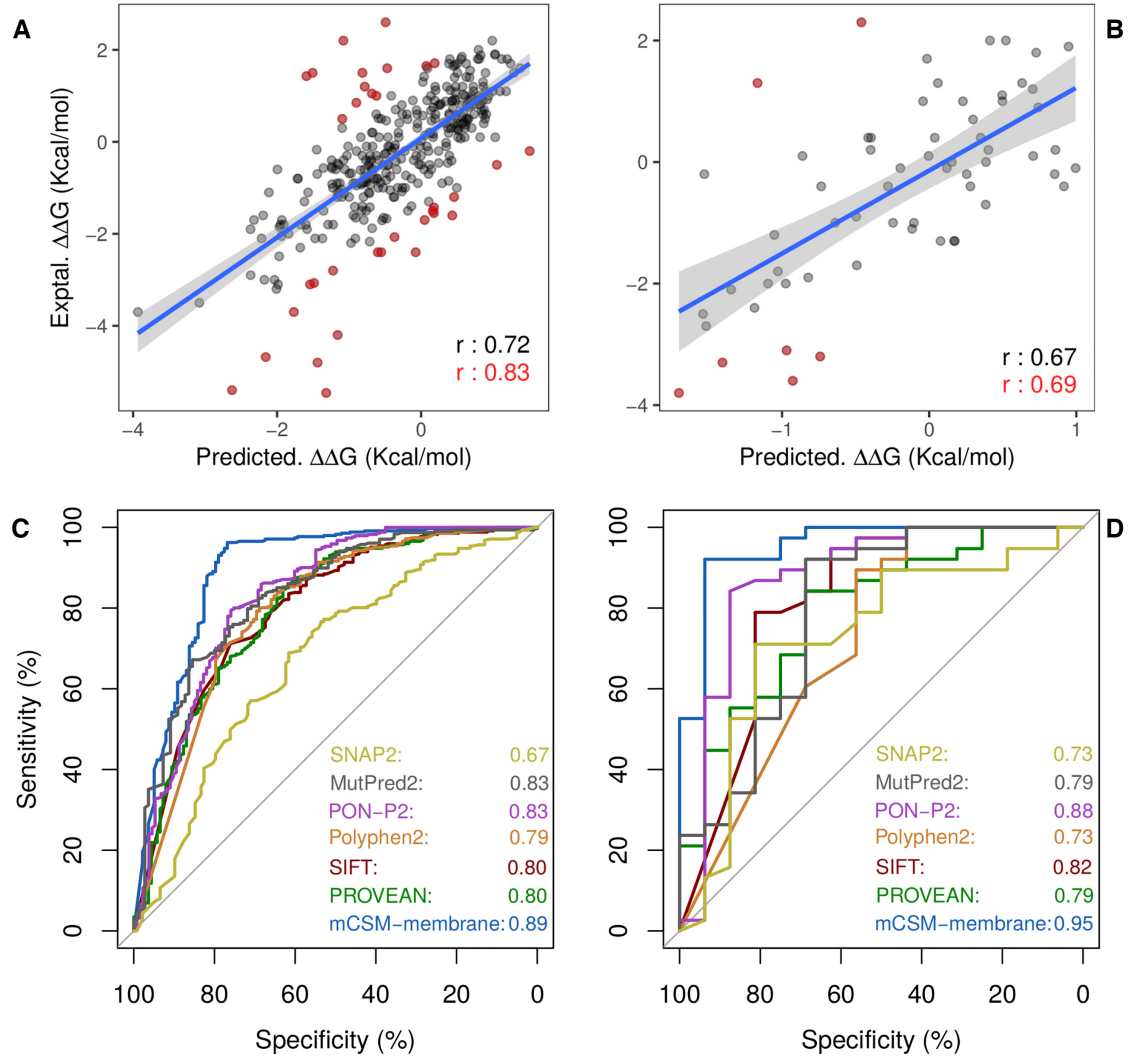
Performance evaluation of mCSM-membrane on cross validation and blind tests. (**A**) shows the performance of mCSM-membrane on predicting effects of mutations on stability for transmembrane proteins during 10-fold cross validation, achieving a Pearson's correlation of 0.72 (0.83 on 90% of the data). During blind test (**B**), mCSM-membrane achieved a correlation of 0.67 with experimental data. For the pathogenicity predictor, (**C**) and (**D**) show the performance of mCSM-membrane in comparison with well-established methods as ROC plots on cross validation and blind test, respectively. Our method achieved AUC of 0.89 and 0.95.

In order to build a robust and reliable model for predicting the effects of mutations on transmembrane stability, mCSM-Membrane was trained using a stratified 10-fold cross-validation approach with 10 bootstrap repetitions. Selection of the blind test was repeated 10 times in a stratified manner, with the model assessed on the remaining data using 10-fold cross-validation, in order to evaluate the robustness of the model. Our method achieved an average Pearson, Spearman and Kendall correlations of 0.72, 0.72 and 0.53, respectively, with a standard deviation of 0.09 across the 10 runs (Figure 2A). We then evaluated the ability of the model to capture destabilizing and stabilizing mutations, using a classification by regression approach. mCSM-Membrane achieved a Mathew's Correlation Coefficient of 0.65 and F1-score of 0.81, correctly capturing 82% of stabilizing and 83% of destabilizing mutations. The effect of considering reverse mutations in the data set was also assessed. When only forward mutations are considered (i.e. removing reverse mutations from training and test sets), performance drops considerably, achieving a Pearson's correlation of 0.58 and a Mathew's Correlation Coefficient of 0.79 and *F*1-score of 0.72, highlighting the importance of considering reverse mutations to balance the data set.

mCSM-Membrane was further evaluated using a blind test set of 62 mutations across 3 proteins, not present in our original training data sets. Our model achieved Pearson, Spearman and Kendall correlations of 0.67, 0.62 and 0.45 (Figure 2B), respectively, consistent with training performance, providing confidence in the generalizability and robustness of our model. Despite the low level of similarity between proteins in training and test sets, and to eliminate any potential selection bias while training and validating our method, we also evaluate the process of selecting an independent test set in a bootstrapped manned 100×, and evaluated the performance of the method on cross validation and test set. mCSM-membrane achieved a correlation of 0.68 (sd = 0.02) on 10-fold cross validation and 0.67 (sd = 0.07) on tests, demonstrating the robustness of the method. Additionally, mCSM-Membrane was compared to well established tools designed to predict the effects of mutations on protein stability. mCSM-Membrane significantly outperformed all tools tested (*P* < 0.05 by Fisher *r*-to-*z* transformation test, Table 1). Consistent with previous results, the other stability predictive tools tested were only weakly predictive across these mutations in transmembrane proteins (Table 1).

**Table 1.** Comparative performance of mCSM-membrane across training and test data sets with alternative stability predictors

	Training		Test	
Method	Pearson's correlation	RMSE	Pearson's correlation	RMSE
FoldX	0.48*	1.18	0.57	1.25
iMutant	0.27*	1.29	0.37*	1.41
CUPSAT	0.01*	1.34	0.15*	1.50
AUTOMUTE (RepTree)	0.17*	1.32	0.05*	1.52
AUTODMUTE (SVM)	0.14*	1.33	0.04*	1.52
MAESTRO	0.20*	1.16	0.17*	1.09
SDM	0.01*	1.34	−0.14*	1.51
mCSM	0.21*	1.31	0.59	1.23
DUET	0.18*	1.32	0.47*	1.34
Dynamut	0.31*	1.27	0.62	1.19
mCSM-membrane	0.72	0.93	0.67	1.13

*P-value < 0.05 by Fisher *r*-to-*z* transformation test compared to mCSM-membrane

### Application to homology models

Experimentally solving structures of transmembrane proteins is particularly challenging. The evolution of comparative homology and threading algorithms, however, has allowed for data augmentation for modelled structures at a proteome-scale (47). To assess the performance of mCSM-membrane on homology models, we have generated models using templates with no more than 37% identity for three different proteins, originally selected as the blind test of our stability predictor. Supplementary Table S2 shows the information on templates used in this process.

Performance on blind test using the homology models deteriorates only slightly (*r* = 0.63. Supplementary Figure S6), compared to performance on experimental structures (*r* = 0.68), highlighting the robustness of the model and ability to accurately predict effects of mutations on homology models. This defines a simple guideline for using mCSM-membrane on homology models.

### Identifying pathogenic mutations in transmembrane proteins

The second predictive mode for mCSM-membrane is a predictor capable of accurately distinguishing between pathogenic and benign mutations tailored for transmembrane proteins (Table 2). This predictor was trained and assessed on 10-fold cross validation, with its performance compared to alternative methods available. Our pathogenicity predictor achieved an Mathew's Correlation Coefficient (MCC) of 0.77 and *F*1-score of 0.91 significantly outperforming SIFT (0.43 and 0.85), PolyPhen2 (0.54 and 0.89) PROVEAN (0.48 and 0.85), MutPred2 (0.48 and 0.79), PON-P2 (0.38, 0.71). The only method that achieved a higher performance than mCSM-membrane during cross validation was BORODA-TM (0.87 and 0.96). However, the discrepancy between the reported performance in cross validation and blind test for BORODA-TM (on blind it achieves an MCC of 0.46 and *F*1 of 0.78) is a strong indication of overfitting.

**Table 2.** Performance assessment of mCSM-membrane in predicting pathogenic mutations across training and test data sets, in comparison with alternative methods.

	Training			Test		
Method	AUC	F1	MCC	AUC	F1	MCC
PolyPhen2	0.79	0.79	0.47	0.73	0.75	0.40
SIFT	0.80	0.77	0.43	0.82	0.84	0.63
PROVEAN	0.80	0.79	0.48	0.79	0.75	0.40
SNAP2	0.67	0.70	0.26	0.73	0.66	0.21
MutPred2	0.75	0.79	0.48	0.75	0.82	0.57
PON-P2	0.83	0.71	0.38	0.88	0.78	0.53
BORODA-TM*	- - -	0.96	0.87	- - -	0.78	0.46
mCSM-membrane	0.89	0.91	0.77	0.95	0.89	0.73

*AUC values were not calculated for BORODA-TM as no scores, rankings or class probabilities were available.

Our predictor was further validated via a blind test achieving an MCC of 0.73 and F1-score of 0.89, performance compatible with cross validation, outperforming alternative methods and demonstrating the efficacy of a transmembrane-specific predictor no identifying pathogenic mutations. Figure 2C and D shows the ROC curves comparing the performance of the four methods during cross validation and blind tests, with our predictor achieving an Area Under the ROC Curve (AUC) of 0.89 and 0.95, respectively.

## CONCLUSION

Here, we introduce mCSM-membrane, a web server that uses our graph-based signatures to predict the effects of single-point missense mutations on the stability of transmembrane proteins and the likelihood of them being disease associated. The method represents a significant advance upon our current predictive platform, outperforming previous methods, which had been built using globular soluble proteins.

mCSM-membrane is freely available as user-friendly and easy to use web server at http://biosig.unimelb.edu.au/mcsm_membrane/.

## Supplementary Material

gkaa416_Supplemental_File
