# Supplementary material for: mCSM-membrane: predicting the effects of mutations on transmembrane proteins
Source: Nucleic Acids Res. 2020 May 29;48(W1):W147–53. doi: 10.1093/nar/gkaa416 (PMC7319563; doi:10.1093/nar/gkaa416)
Supplement: gkaa416_Supplemental_File [file gkaa416_supplemental_file.docx]

# **SUPPLEMENTARY MATERIAL**

**mCSM-membrane: predicting the effects of mutations on transmembrane proteins**

Douglas E.V. Pires^1,2,3,^*, Carlos H. M. Rodrigues^1,2^, David B. Ascher^1,2,4,^*

^1^Computational Biology and Clinical Informatics, Baker Institute, Melbourne, Victoria 3004, Australia

^2^Department of Biochemistry and Molecular Biology, Bio21 Institute, University of Melbourne, Parkville, VIC, 3052, Australia

^3^School of Computing and Information Systems, University of Melbourne, Parkville, VIC, 3052, Australia

^4^Department of Biochemistry, University of Cambridge, Cambridge, CB2 1GA, UK

*To whom correspondence should be addressed D.B.A. Tel: +61 90354794; Email: [david.ascher@unimelb.edu.au](mailto:david.ascher@unimelb.edu.au). Correspondence may also be addressed to D.E.V.P. [douglas.pires@unimelb.edu.au](mailto:douglas.pires@unimelb.edu.au).


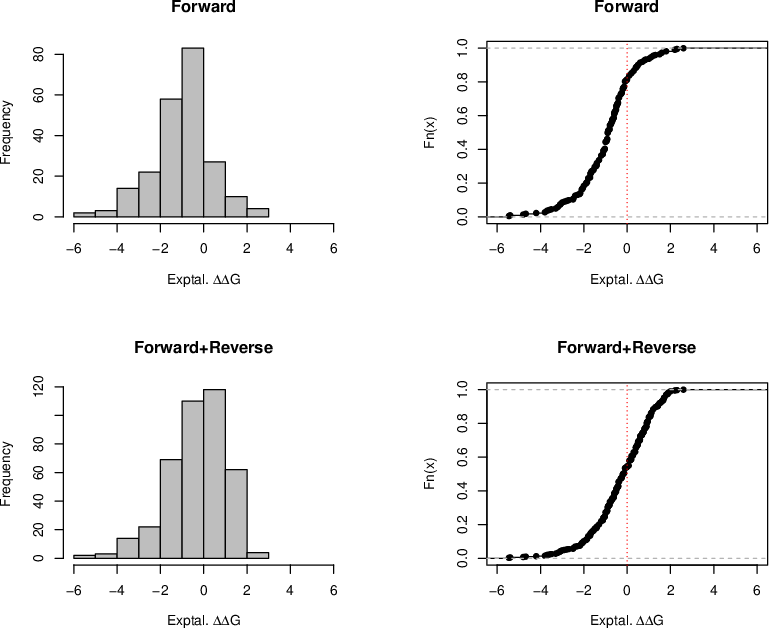


**Figure S1**. Distribution of experimental effects of mutations for the stability data set. The top plots show a histogram (on the left) and a cumulative distribution (on the right) of effects of mutations in transmembrane protein stability (given in Kcal/mol) for forward mutations. The bottom plots show the difference in distribution once reverse mutations are modelled and included in the dataset, making it considerably more balanced.


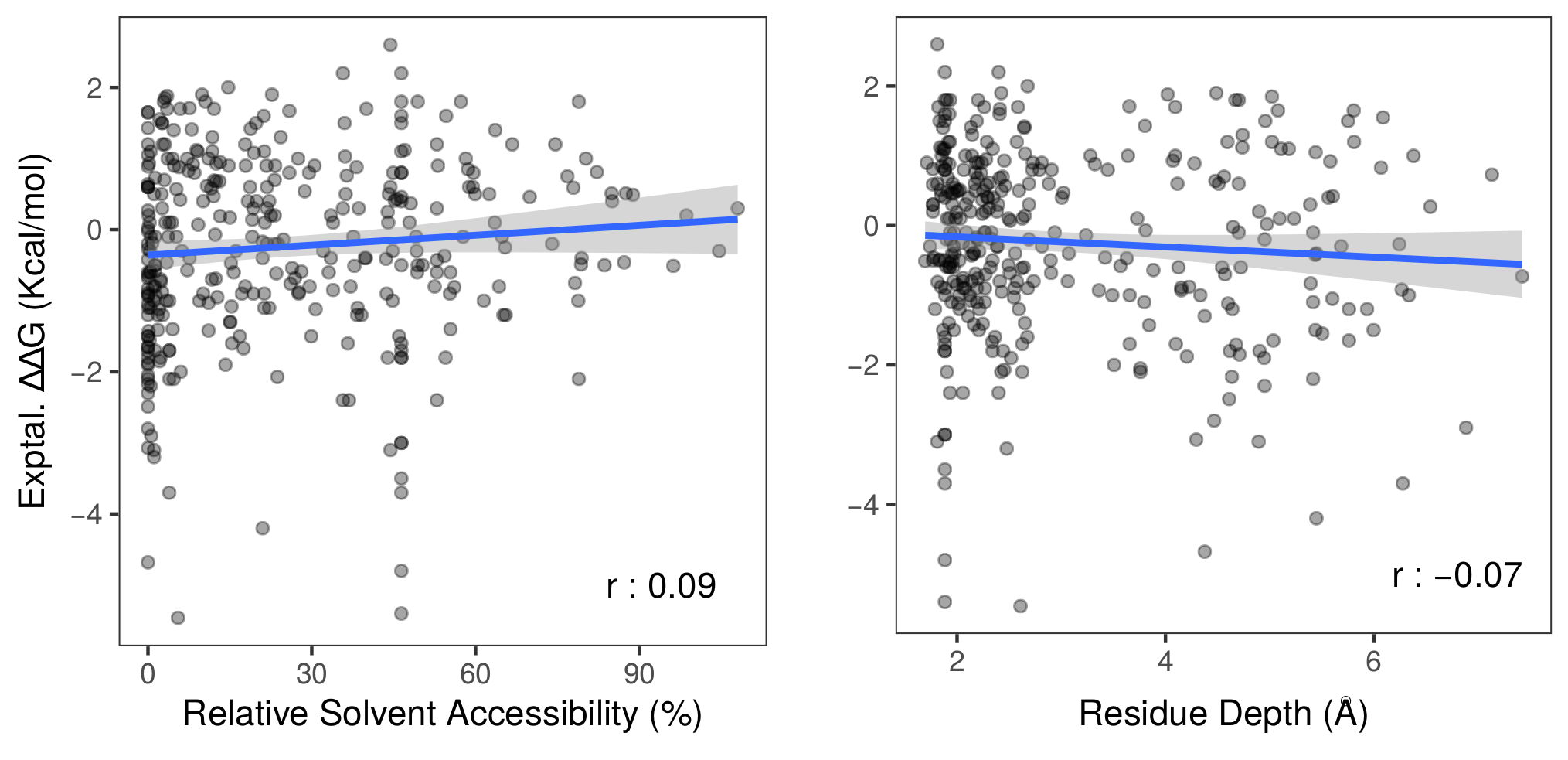


**Figure S2**. Correlation between residue depth and solvent accessible area with experimentally measured effects of mutations on stability of transmembrane proteins. Differently from globular proteins, no correlation was identified.


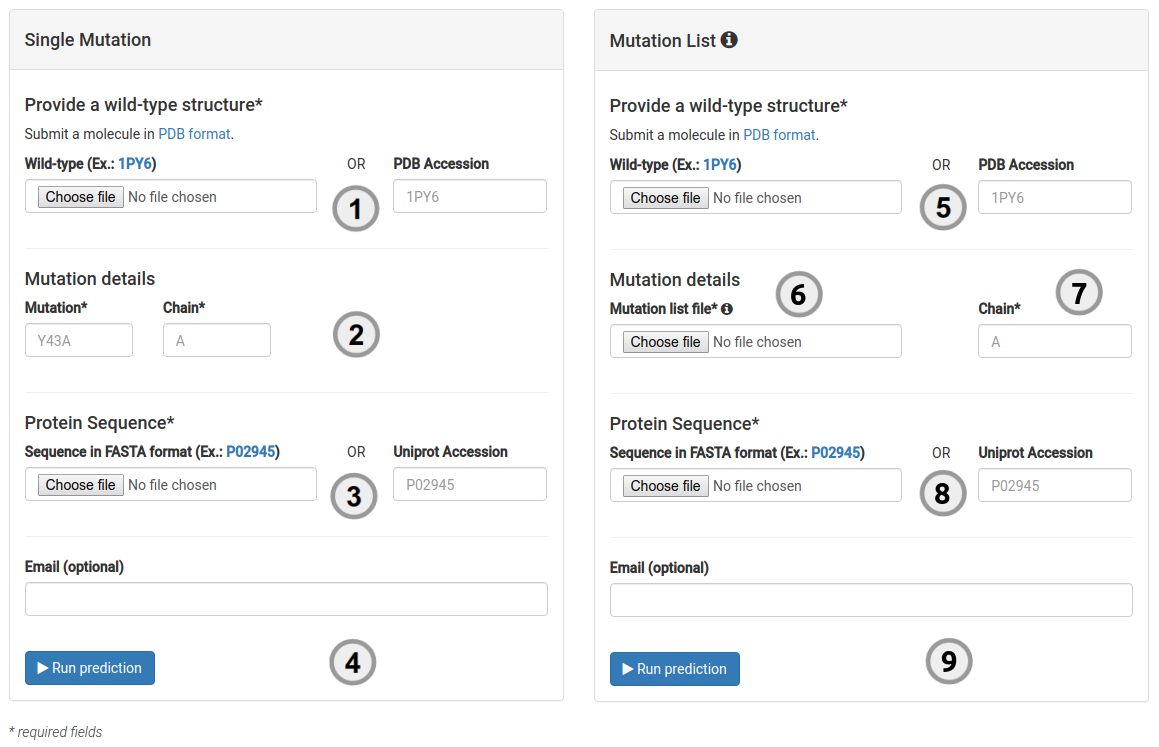


**Figure S3**. Submission form for mCSM-membrane. For both prediction in protein stability and the pathogenicity, users have two main options. For “Single Mutation”, users are prompted to provide a protein structure in PDB format, by either uploading it or providing a PDB accession code (1/5), a mutation code and chain ID (2) and the Uniprot  accession code for the protein (3) and then click in “Run prediction” (4). For “Mutation List”, instead of providing a single mutation, users are prompted to provide a text file with a list of mutations (6), one per line, the chain ID (7) and Uniprot accession (8) before submitting a job (9).


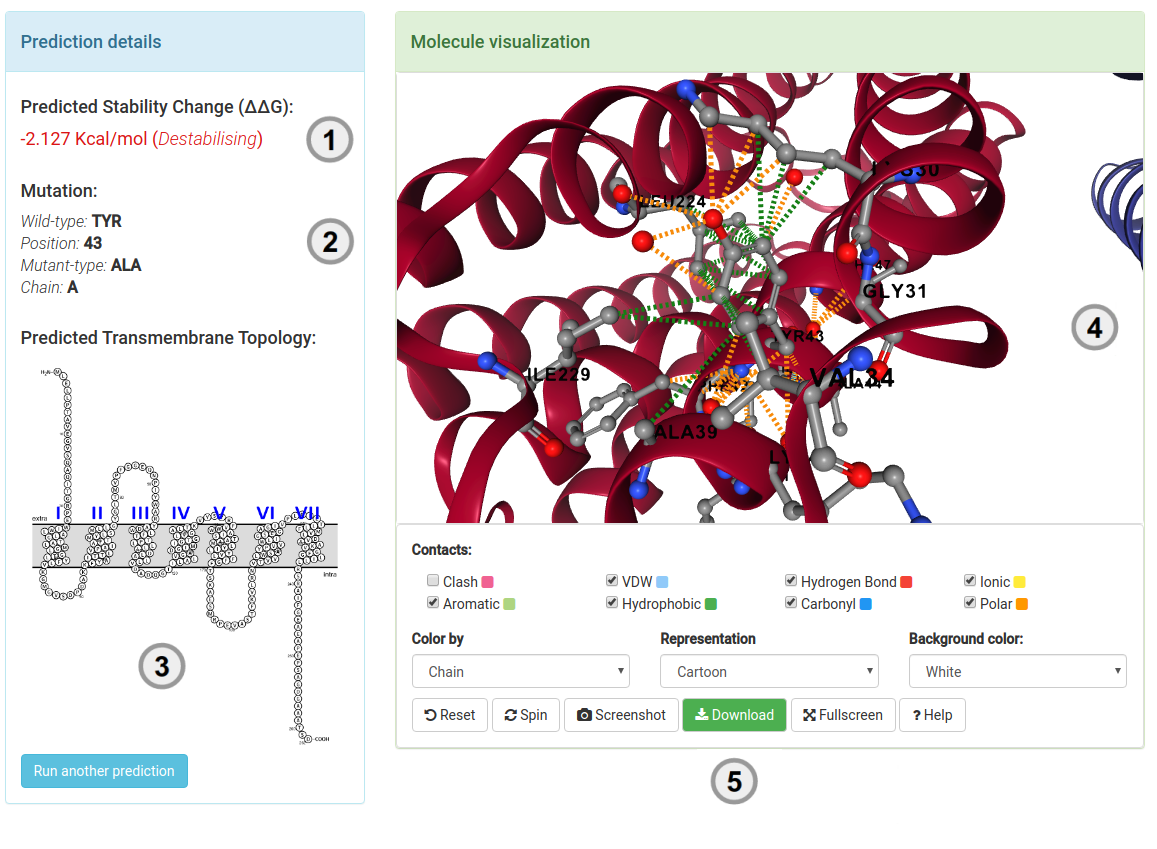
**Figure S4**. Results page for “Single Mutation” prediction. mCSM-membrane will output the prediction (1) as either “Pathogenic/Benign” for the pathogenicity predictor or the the change in stability (in kcal/mol) for the stability predictor. A summary of the mutations is also shown (2), together with a depiction of the predicted transmembrane topology (3). An interactive molecule visualization depicting the wild-type residue environment is also shown (4) and interatomic interactions made by mutated residue are available for download as a Pymol session file (5).


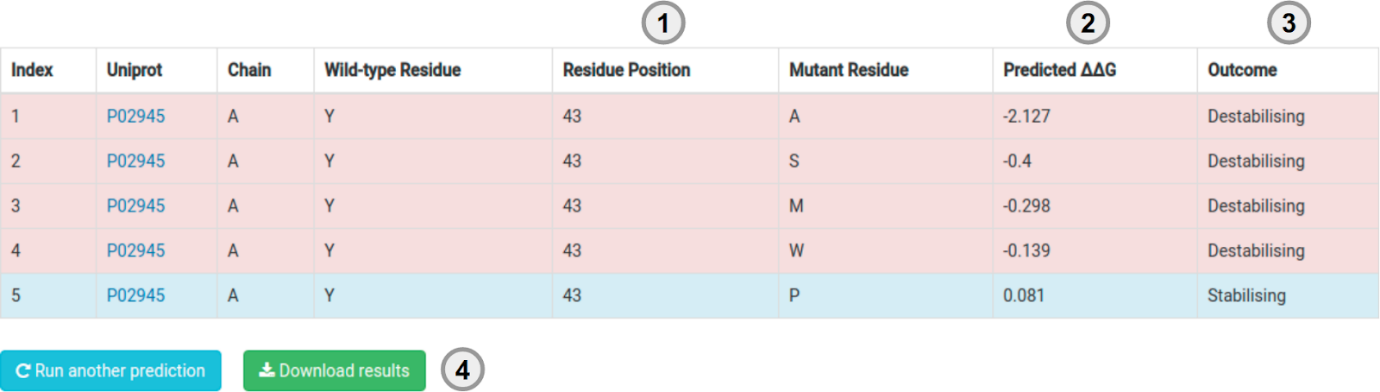


**Figure S5**. Results page for “Mutation List”. For both prediction options (stability and pathogenicity) the results are shown in tabular format, including information about the mutation (1) and the predicted outcome (2/3). The predictions can also be downloaded as a tab-separated file.


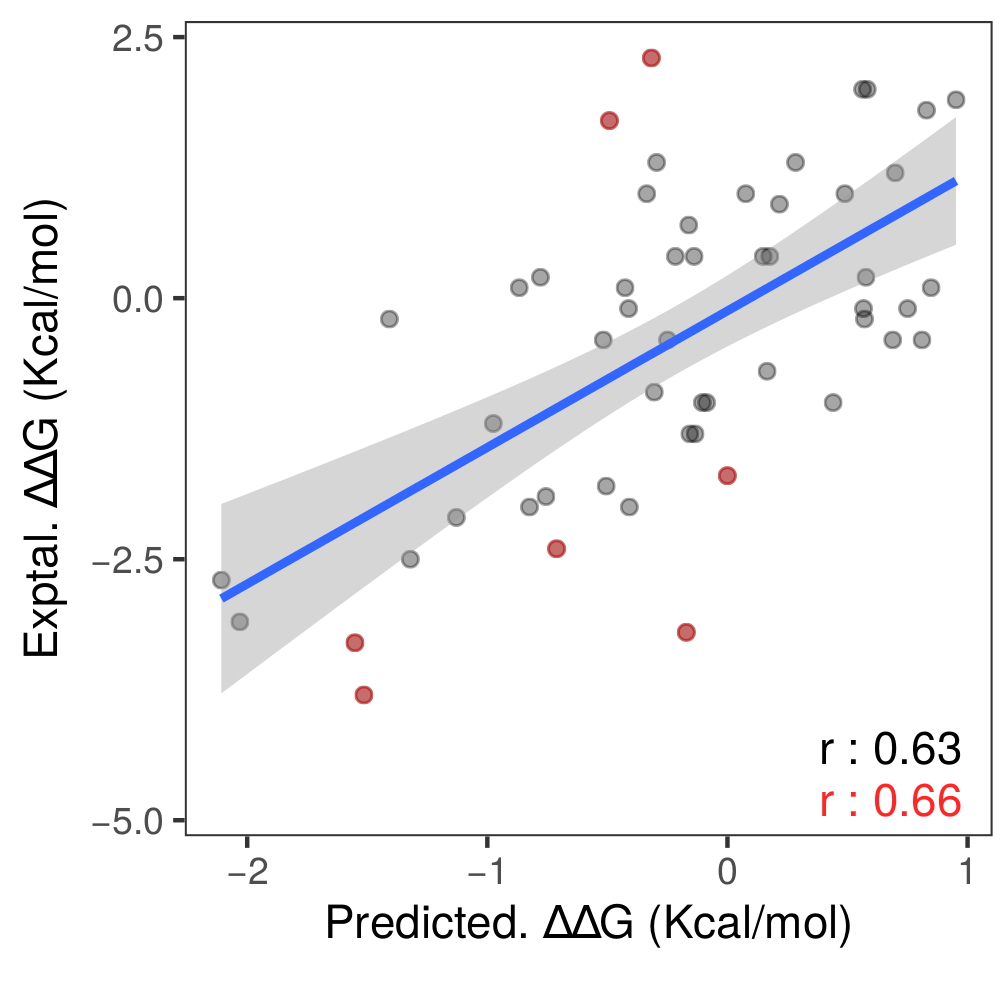


**Figure S6**. Performance evaluation of mCSM-membrane on homology models. The method was capable of accurately predicting effects of mutations on stability for transmembrane proteins generated using homology models with low-identity templates, achieving a Pearson’s correlation of 0.63 (0.66 on 90% of the data).

**Table S1.** Pairwise protein identity levels (%) for proteins used to train and test mCSM-membrane stability predictor.

|  | **1AFO** | **3GP6** | **1PY6** | **2XOV** | **2K73** | **1QJP** | **1QD6** |
| --- | --- | --- | --- | --- | --- | --- | --- |
| **1AFO** |  | 0.5 | 3.6 | 5.4 | 3.8 | 7.0 | 2.1 |
| **3GP6** |  |  | 6.4 | 7.4 | 2.5 | 12.3 | 15.7 |
| **1PY6** |  |  |  | 9.7 | 11.8 | 2.3 | 4.0 |
| **2XOV** |  |  |  |  | 14.3 | 13.0 | 11.6 |
| **2K73** |  |  |  |  |  | 0.6 | 1.5 |
| **1QJP** |  |  |  |  |  |  | 9.9 |
| **1QD6** |  |  |  |  |  |  |  |

**Table S2.** Homology modeling Pairwise protein identity levels (%) for proteins used to train and test mCSM-membrane stability predictor.

| **Protein** | **UNIPROT** | **Template** | **Template identity** |
| --- | --- | --- | --- |
| Glycophorin A | P02724 | 5ZAZ | 28% |
| OmpA | P0A910 | 1QJ8 | 23% |
| DsbB | P0A6M2 | 3KP9 | 9% |
